# Prevalence and associated factors of screening-defined myopia among schoolchildren in Urumqi: a comparison of two cross-sectional surveys conducted in 2012 and 2019

**DOI:** 10.3389/fpubh.2026.1875125

**Published:** 2026-08-21

**Authors:** Jin Luo, Xuemei Li, Haonan Shi, Jia Zhu, Jin Wang, Xuan Yao, Tingting Wang

**Affiliations:** 1 School of International and Public Affairs, Institute of Health Yangtze River Delta Research, Shanghai Jiao Tong University, Shanghai, China; 2 Department of Ophthalmology, Urumqi Traditional Chinese Medicine Hospital, Urumqi, China; 3 Mental Health Center Affiliated to School of Medicine, Shanghai University (Shanghai Hongkou Mental Health Center), Shanghai, China; 4 School of Medicine, Shanghai University, Shanghai, China; 5 The Third Ward of the Medical Staff Health Care Center of Xinjiang Uygur Autonomous Region People's Hospital, Urumqi, China; 6 Affiliated Zhoupu Hospital, Shanghai University of Medicine & Health Sciences, Shanghai, China; 7 School of Nursing & Health Management, Shanghai University of Medicine & Health Sciences, Shanghai, China; 8 Department of Nursing, Pudong Gongli Hospital, Shanghai University of Medicine & Health Sciences, Shanghai, China

**Keywords:** a cross-sectional survey, myopia, prevalence, risk factors, schoolchildren

## Abstract

**Background:**

Myopia is an important public health concern among schoolchildren. This study described the prevalence of screening-defined myopia and its associated factors among schoolchildren in Urumqi using data from two independently sampled cross-sectional surveys conducted in 2012 and 2019.

**Methods:**

The 2012 survey included 1,938 students from Xinshi and Shayibake districts, whereas the 2019 survey included 9,503 students from Xinshi, Shayibake, Tianshan, and Shuimogou districts. Information on visual status and individual and environmental factors was collected. Crude and internally standardised prevalence estimates were calculated. Because the two surveys differed in geographic coverage and sample composition, comparisons between survey years were interpreted descriptively.

**Results:**

The crude prevalence of myopia was 39.7% in the 2012 survey and 37.1% in the 2019 survey. After equal-weight standardisation by school stage, the corresponding estimates were 45.9 and 43.7%, respectively. Descriptive prevalence patterns differed across school stages; however, because the two surveys did not have identical geographic coverage or sampling frames, these differences should not be interpreted as evidence of a population-level temporal trend. Myopia was higher in females than males and lower in Han than other ethnic groups (all *p* < 0.05). Multivariable logistic regression for 2012: risk factors - reading <30 cm from books (*OR* = 1.51, 95%*CI* = 1.28–1.78), poor reading/writing habits (*OR* = 1.55, 95%*CI* = 1.26–1.91), daily computer use (*OR* = 1.21, 95%*CI* = 1.00–1.46), parental myopia (*OR* = 2.11, 95%*CI* = 1.65–2.71); protective factors - eye exercises (*OR* = 0.66, 95%*CI* = 0.53–0.81), daily outdoor time (*OR* = 0.84, 95%*CI* = 0.71–1.00), daily sleep time (*OR* = 0.75, 95%*CI* = 0.60–0.95). For 2019: risk factors - reading <30 cm (*OR* = 1.69, 95%*CI* = 1.56–1.82), poor habits (*OR* = 1.22, 95%*CI* = 1.09–1.36), parental myopia (OR = 2.22, 95%CI = 2.03–2.43); protective factors—resting eyes after 1 h reading/writing (*OR* = 0.79, 95%*CI* = 0.74–0.84), eye exercises (*OR* = 0.87, 95%*CI* = 0.79–0.95), daily TV hours (*OR* = 0.85, 95%*CI* = 0.79–0.92), frequent distance viewing (*OR* = 0.85, 95%*CI* = 0.74–0.98), daily outdoor time (*OR* = 0.90, 95%*CI* = 0.83–0.98), daily sleep time (*OR* = 0.84, 95%*CI* = 0.75–0.94), weekly frequency of eating zhuafan (*OR* = 0.85, 95%*CI* = 0.77–0.95).

**Conclusion:**

Myopia represented a substantial public health burden among schoolchildren in both surveys. Several behavioural and familial characteristics were associated with myopia, although the cross-sectional design precluded causal inference. Because the two surveys used different sampling frames, the observed between-survey differences should not be interpreted as definitive evidence of a temporal change in population prevalence.

## Background

Myopia has become a public health problem over the world and is a major ocular disease that impairs vision in adolescents. In 2012, Morgan IG, an Australian scholar, in the lancet journal (1) stated that students in Southeast Asia are currently the most myopic, and focused on China, Korea, and Japan. It is known from research investigations abroad (2) that currently about 28.3% of people worldwide suffer from myopia. It is predicted that the global myopia rate will rise to 50% and the total myopic population will reach 4,758 million in the next 30 years (3). Data from the Korean national health and Nutrition Examination Survey (4) from 2008 to 2012 showed that the prevalence of high myopia in the adult population was 7.0%. Importantly, the prevalence of high myopia in the adult population increased to 8.0% according to the Korean national health and Nutrition Examination Survey data from 2013 to 2014 (5). In addition, the incidence and rate of progression of myopia are higher in China compared to other Asian countries (6), and the population most affected by myopia is schoolchildren. A 2012 survey of myopia among schoolchildren and adolescents from 6 provinces and municipalities in China by the Peking University Institute of child and adolescent health (7) concluded that the prevalence of myopia among schoolchildren was 55.7% across 6 provinces in China. A cohort study conducted by Ma et al. (8) involving 1- to 3-year students in Baoshan, Shanghai, China, showed that the prevalence of myopia in all grades at the initial stage of the study was 7.4, 15.2, 25.9%, and rose to 30.0, 29.2, 33.2% at the first follow-up visit after 2 years. Therefore, prevention and control of myopia among schoolchildren is urgent at present.

Xinjiang is a multiethnic cluster, and there are large differences in living dietary habits among the ethnic groups. Although this difference makes the myopia prevalence of minorities in Xinjiang lower than that of Han, and also makes the overall myopia prevalence of schoolchildren in Xinjiang lower than the national level, according to previous studies, the myopia prevalence in Xinjiang is also increasing year by year. Tang et al. (9) collected the data repository of physical health survey of elementary and middle school students in Urumqi of Xinjiang region showed that the percentage of poor vision of the 3 survey for 7- to 18-year-old students in 2006, 2011 and 2016 was 26.68, 30.73 and 36.00%, respectively, and most of them had poor vision condition of myopia. Our subject group also previously conducted a survey on myopia among secondary students in Urumqi compared with that in Yining of Xinjiang (10, 11), the results showed that in 2013, the overall myopia rate among secondary students in Urumqi was 60.12%; in 2016, the overall myopia rate among elementary and secondary students in Yining was 38.25%. In 2018, in order to fully follow the spirit of the implementation of the party’s nineteenth spirit and the national education assembly, the Ministry of Education decided to select and build a number of pilot counties (cities and districts) for national childhood and adolescent myopia prevention and control reform and a pilot region for national childhood and adolescent myopia prevention and control reform, and Urumqi is the pilot region for myopia prevention and control reform in Xinjiang (12, 13).

We conducted a survey and study on myopia among primary and secondary students in Urumqi in September 2012 and December 2019, respectively. The results of this study are necessary to understand the changing situation of myopia among schoolchildren in Urumqi and provide a theoretical basis for prevention and control.

### Subjects

Two independent school-based cross-sectional surveys were conducted in Urumqi in September 2012 and December 2019. In 2012, students were recruited from Xinshi and Shayibake districts. A total of 2,400 questionnaires were distributed, of which 1,938 were valid, corresponding to a response rate of 80.8%. In 2019, stratified cluster sampling was used to recruit students from 11 schools in Xinshi, Shayibake, Tianshan, and Shuimogou districts. Students from the third grade of primary school to the second grade of senior high school were eligible to participate. Of the 10,000 questionnaires distributed, 9,503 were valid, corresponding to a response rate of 95.0%.

The two surveys differed in geographic coverage, sample size, and response rate and should therefore be regarded as independent cross-sectional samples rather than repeated observations of an identical source population. The study was approved by the Ethics Committee of the First Affiliated Hospital of Xinjiang Medical University, and informed consent was obtained before participation.

## Methods of investigation

The questionnaire was self-designed. The design of the main content of the questionnaire, in principle, was carried out according to the requirements of the Ministry of Education issued a working protocol for myopia prevention and control among primary and secondary students. The content of the questionnaire included the following 7 broad categories: (1) Demographic data, including gender, ethnicity, date of birth, and school attended, etc.; (2) Visual acuity and genetic family circumstances, including whether they had myopia, eyeglasses replacement status, and the presence of myopia in the parents, paternal/maternal grandparents, and siblings; (3) Eye habit behaviors, including reading and writing habits, sleeping time, TV watching time, mobile phone/tablet/computer usage, etc.; (4) Academic problems, including academic performance, learning stress, physical education and frequency of extracurricular activities, etc.; (5) Dietary factors, including the number of meals, fond of food, and dietary bias situations; (6) The lighting conditions of the classroom, including the students’ satisfaction with the natural light and the electric light of the classroom and the effect on reading and writing, etc.; (7) Other factors, including face washing, mode of school, whether lights were turned on while sleeping, etc.

The questionnaire was filled out by the students themselves. At least one trained school physician and two subject investigators answered the questions for the students during the survey. Questionnaires were reviewed by school physicians and investigators and excluded for ineligibility.

### Determination of relevant outcomes

(1) Definition of screening-defined myopia: Uncorrected distance visual acuity was assessed by trained school physicians and investigators using the standard logarithmic visual acuity chart in accordance with the protocol of the Chinese National Survey on Students’ Constitution and Health (14). Uncorrected distance visual acuity ≥5.0 on the five-point logarithmic scale was considered normal, whereas visual acuity <5.0 in either eye was considered reduced. Students with reduced visual acuity underwent pupil dilation with tropicamide and an ophthalmic examination by the same attending ophthalmologist using slit-lamp biomicroscopy and direct ophthalmoscopy. Clinically identifiable alternative causes of reduced visual acuity, including hyperopia, amblyopia, strabismus, ocular trauma, and glaucoma, were excluded. In the absence of cycloplegic refraction and spherical equivalent measurements, participants with uncorrected distance visual acuity <5.0 in at least one eye and no identified alternative ocular condition were classified as having screening-defined myopia. When visual acuity differed between the two eyes, participant-level classification was based on the worse eye. This operational definition was used consistently in both surveys. (2) Determination of frequency: “often “refers to more than half of the time exposed to the factor in question or at least 2 exposures to the factor in 1 week during a certain activity. “occasionally “refers to less than half of the time with exposure to the factor in question or 1 week with less than 2 exposures higher than once in 2 weeks. “no “refers to never exposure to the factor in question. (3) Bad reading and writing habits: lying down to see a mobile phone or reading; the Grip pen site is too lower end or finger blocks one eye of sight; body or head skewing when writing; read a book when walking or travelling.

### Statistical analysis

Data were entered using EpiData version 3.1 and analysed using SPSS version 26.0. Categorical variables were summarised as frequencies and percentages and compared using Pearson’s *χ*^2^ test. Crude prevalence estimates were calculated separately for the 2012 and 2019 surveys. For descriptive purposes, internally standardised prevalence estimates were calculated using a hypothetical standard population in which equal weights of one third were assigned to each of the three prespecified age groups (<12, 12–15, and >15 years) and, separately, to each of the three school stages. Candidate behavioural and environmental variables were initially examined in year-specific univariable analyses. Variables associated with screening-defined myopia at a two-sided *p-*value <0.05 were entered into the corresponding year-specific multivariable unconditional logistic regression model. Sex, ethnicity, age group, and school stage were specified *a priori* as adjustment covariates and were included in both models irrespective of their univariable statistical significance. Adjusted associations were reported as ORs with 95% CIs. Model goodness of fit was assessed using the Hosmer–Lemeshow test. All statistical tests were two-sided, with *α* = 0.05.

## Results

### General condition of investigated subjects

A total of 1938 schoolchildren were surveyed in 2012, of whom 852 (44.0%) were boys and 1,086 (56.0%) were girls. The maximum age was 19.50 years, the minimum was 8.83 years, and the mean age was (13.72 ± 2.58) years. There were 868 (44.8%) Han ethnicity, and 1,070 (55.2%) other ethnicities. Primary school students were 490 (25.3%), junior high school students were 1,214 (62.6%), and senior high school students were 234 (12.1%).

A total of 9,503 schoolchildren in Urumqi were surveyed in 2019, of whom 4,774 (50.2%) were boys and 4,729 (49.8%) were girls. The maximum age was 20.13 years, the minimum age was 8.95 years, and the mean age was (12.62 ± 2.86) years. There were 4,649 (48.9%) Han ethnicity, and 4,854 (51.1%) other ethnicities. Primary school students accounted for 4,180 participants (44.0%), junior high school students for 3,717 (39.1%), and senior high school students for 1,606 (16.9%).

### Characteristics of myopia among primary and secondary school students in Urumqi in 2012

In the 2012 survey, 770 of the 1,938 participants were classified as having myopia, corresponding to a crude prevalence of 39.7% (Table 1). Using equal weights across the three prespecified age groups, the internally age-standardised prevalence was 38.2%. Using the same equal-weight approach across the three school stages, the school-stage-standardised prevalence was 45.9%. Among them, female students had higher myopia rate than male students (*p* < 0.05); The myopia rate was higher in Han than in other ethnic groups (*p* < 0.05); The myopia rates of students aged over 15 years old and high school were higher than those of the remaining groups (all *p* < 0.05).

**Table 1:** Prevalence of myopia among primary and secondary school students in Urumqi in 2012 (*n* = 1938).

Characteristic	Category	Total, *n*	Screening-defined myopia, *n*	Prevalence (%)	*χ* ^2^	*p* value
Gender	Male	852	280	32.9	29.05	<0.01
	Female	1,086	490	45.1		
Ethnicity	Han	868	488	56.2	178.52	<0.01
	Others	1,070	282	26.4		
Age (years)	<12	503	58	11.5	301.82	<0.01
	12–15	836	335	40.1		
	>15	599	377	62.9		
Education	Primary school	490	52	10.6	371.79	<0.01
	Secondary school	1,214	521	42.9		
	High school	234	197	84.2		

### Characteristics of myopia among primary and secondary school students in Urumqi in 2019

In the 2019 survey, 3,525 of the 9,503 participants were classified as having myopia, corresponding to a crude prevalence of 37.1% (Table 2). The equal-weight age-standardised prevalence was 44.6%, and the equal-weight school-stage-standardised prevalence was 43.7%. Among them, female students had higher myopia rate than male students (*p* < 0.05); The myopia rate was higher in Han than in other ethnic groups (*p* < 0.05); The myopia rates of students aged over 15 years old and high school were higher than those of the remaining groups (all *p* < 0.05).

**Table 2:** Prevalence of myopia among primary and secondary school students in Urumqi in 2019 (*n* = 9,503).

Characteristic	Category	Total, *n*	Screening-defined myopia, *n*	Prevalence (%)	*χ* ^2^	*p* value
Gender	Male	4,774	1,545	32.4	92.01	<0.01
	Female	4,729	1980	41.9		
Ethnicity	Han	4,649	2,361	50.8	731.20	<0.01
	Others	4,854	1,164	24.0		
Age (years)	<12	4,714	979	20.8	1195.57	<0.01
	12–15	3,332	1,598	48.0		
	>15	1,457	948	65.1		
Education	Primary school	4,180	764	18.3	1392.53	<0.01
	Secondary school	3,717	1,667	44.8		
	High school	1,606	1,094	68.1		

### Univariate analysis of myopia among schoolchildren in Urumqi between 2012 and 2019

The result of this survey (Table 3) showed that in 2012, academic performance, students’ eyes when reading and writing were less than 30 cm away from books, poor reading and writing habits, lights on when the reading and writing environment is darker, eyes rest after 1 h of reading and writing time, eye exercises, reading and writing duration, hours of computer use per day, frequently looking into distance, time spent outdoors per day, time spent sleeping per day, ways of washing face, and numbers of eating zhuafan per week were the main environmental factors affecting the incidence of myopia among schoolchildren (all *p* < 0.05). In 2019, academic performance, students’ eyes when reading and writing were less than 30 cm away from books, poor reading and writing habits, lights on when the reading and writing environment is darker, eyes rest after 1 h of reading and writing time, eye exercises, reading and writing duration, hours of computer use per day, hours of watching television per day, frequently looking into distance, time spent outdoors per day, time sleeping per day, and numbers of eating zhuafan per week were the main environmental factors affecting the incidence of myopia among schoolchildren (all *p* < 0.05).

**Table 3:** Univariate analysis of myopia among schoolchildren in Urumqi in 2012 and 2019 (*n* = 9,503).

Factor	Category	2012				2019			
		Under each survey year: Screening-defined myopia, *n*	Prevalence (%)	*χ^2^*	*p* value	Under each survey year: Screening-defined myopia, *n*	Prevalence (%)	*χ* ^2^	*p* value
Academic performance	Excellent	90	22.6	62.15	<0.01	1,379	34.5	32.73	<0.01
	Mid upper	175	43.2			616	36.7		
	Mid	413	44.6			940	38.4		
	Mid lower	64	44.1			411	43.0		
	Poor	28	43.8			179	42.3		
Students’ eyes when reading and writing were less than 30 cm away from books	Often	347	52.3	137.34	<0.01	1,141	53.5	605.04	<0.01
	Occasionally	179	23.6			1896	39.3		
	No	244	47.2			488	19.2		
Poor reading and writing habits	Often	249	67.8	302.87	<0.01	2,595	41.9	263.18	<0.01
	Occasionally	428	45.8			810	32.7		
	No	93	14.6			120	14.5		
Lights on when the reading and writing environment is darker eyes rest after 1 h of reading and writing time	Often	348	41.4	39.57	<0.01	1723	39.5	119.86	<0.01
	Occasionally	324	45.1			978	42.5		
	No	98	25.9			824	29.0		
	Often	264	33.2	25.53	<0.01	655	21.6	467.74	<0.01
	Occasionally	358	45.5			1,485	42.9		
	No	148	41.6			1,385	46.0		
Eye exercises	Do daily	390	31.0	125.92	<0.01	2,330	33.5	172.59	<0.01
	Do sometimes	303	53.1			1,102	48.7		
	Never	77	70.6			93	32.4		
Reading and writing duration	≤1 h	120	33.1	8.31	0.02	350	22.7	316.38	<0.01
	1-2 h	439	41.3			1,391	33.4		
	>2 h	211	41.3			1784	46.9		
Hours of computer use per day	≤1 h	433	32.3	101.71	<0.01	3,248	38.1	38.32	<0.01
	1-2 h	268	56.9			177	29.2		
	>2 h	69	55.2			100	26.5		
Hours of watching television per day	≤1 h	488	39.7	2.77	0.25	2,404	44.3	290.86	<0.01
	1-2 h	214	41.7			819	29.0		
	>2 h	68	34.9			302	24.2		
Frequently looking into distance	No	233	50.9	31.09	<0.01	620	39.6	5.21	0.02
	Yes	537	36.3			2,905	36.6		
Time spent outdoors per day	≤1 h	317	35.4	13.92	<0.01	2,876	40.5	145.63	<0.01
	1-2 h	332	44.4			427	29.2		
	>2 h	121	41.0			222	23.7		
Time sleeping per day	≤8 h	559	46.1	55.95	<0.01	2,877	43.9	420.64	<0.01
	8-10 h	206	29.5			608	22.1		
	>10 h	5	18.5			40	19.7		
Ways of washing face	Flowing water	523	37.8	14.09	<0.01	2,965	37.4	2.22	0.33
	Pot wash	88	52.7			213	35.3		
	Both them	159	41.0			347	35.3		
Numbers of eating zhuafan per week	≤2 times/week	704	45.5	107.57	<0.01	2,490	43.7	263.14	<0.01
	2 times/week	66	16.8			1,035	27.2		

### Multivariate logistic regression analysis of myopia among schoolchildren in Urumqi

The results of the year-specific multivariable logistic regression analyses are presented in Table 4. Candidate behavioural and environmental variables with a two-sided *p-*value <0.05 in the corresponding univariable analysis were entered into each model. Sex, ethnicity, age group, and school stage were included as prespecified adjustment covariates regardless of their univariable statistical significance. Screening-defined myopia was specified as the dependent variable (0 = no; 1 = yes). In 2012, a reading distance of less than 30 cm, poor reading and writing habits, longer daily computer use, and parental myopia were associated with higher odds of myopia. Performing eye exercises, spending more time outdoors, and having a longer sleep duration were associated with lower odds of myopia. In 2019, a reading distance of less than 30 cm, poor reading and writing habits, and parental myopia were associated with higher odds of myopia, whereas resting the eyes after 1 h of reading or writing, performing eye exercises, watching television for longer periods, frequently looking into the distance, spending more time outdoors, having a longer sleep duration, and eating zhuafan more frequently were associated with lower odds of myopia. The Hosmer–Lemeshow tests showed no statistically significant evidence of lack of fit for either multivariable model (2012: *p* = 0.078; 2019: *p* = 0.135).

**Table 4:** Year-specific multivariable logistic regression analyses of factors associated with screening-defined myopia^*^.

Factor	Reference category	2012		2019	
		Under each survey year: Adjusted *OR* (95% *CI*)	*p* value	Under each survey year: Adjusted *OR* (95% *CI*)	*p* value
Academic performance	Poor	1.04 (0.92 ~ 1.17)	0.58	1.02 (0.98 ~ 1.06)	0.36
Students’ eyes when reading and writing were less than 30 cm away from books	Almost none	1.51 (1.28 ~ 1.78)	<0.01	1.69 (1.56 ~ 1.82)	<0.01
Poor reading and writing habits	Almost none	1.55 (1.26 ~ 1.91)	<0.01	1.22 (1.09 ~ 1.36)	<0.01
Lights on when the reading and writing environment is darker	Almost none	1.07 (0.92 ~ 1.26)	0.39	1.00 (0.93 ~ 1.08)	0.97
Eyes rest after 1 h of reading and writing time	Almost none	0.88 (0.74 ~ 1.05)	0.15	0.79 (0.74 ~ 0.84)	<0.01
Eye exercises	Never	0.66 (0.53 ~ 0.81)	<0.01	0.87 (0.79 ~ 0.95)	<0.01
Reading and writing duration	≤1 h	1.07 (0.89 ~ 1.29)	0.47	1.07 (0.99 ~ 1.16)	0.08
Hours of computer use per day	≤1 h	1.21 (1.00 ~ 1.46)	0.05	0.90 (0.79 ~ 1.01)	0.08
Hours of watching television per day	≤1 h	—	—	0.85 (0.79 ~ 0.92)	<0.01
Frequently looking into distance	No	0.88 (0.67 ~ 1.16)	0.36	0.85 (0.74 ~ 0.98)	0.03
Time spent outdoors per day	≤1 h	0.84 (0.71 ~ 1.00)	0.05	0.90 (0.83 ~ 0.98)	0.02
Time sleeping per day	≤8 h	0.75 (0.60 ~ 0.95)	0.02	0.84 (0.75 ~ 0.94)	<0.01
Ways of washing face	Flowing water	0.94 (0.82 ~ 1.08)	0.37	—	—
Numbers of eating zhuafan per week	≤2	0.76 (0.51 ~ 1.07)	0.11	0.85 (0.77 ~ 0.95)	<0.01
Parental myopia	At least one myopic parent vs. neither parent	2.11 (1.65 ~ 2.71)	<0.01	2.22 (2.03 ~ 2.43)	<0.01
Myopia in parent/maternal grandparents	No	0.86 (0.68 ~ 1.08)	0.19	1.03 (0.89 ~ 1.19)	0.71

^*^OR, odds ratio; CI, confidence interval. Candidate behavioural and environmental variables associated with screening-defined myopia at *p* < 0.05 in the corresponding univariable analysis were entered into the year-specific multivariable model. Both models were additionally adjusted for sex, ethnicity, age group, and school stage, which were included *a priori* irrespective of their univariable statistical significance. Model goodness of fit was assessed using the Hosmer–Lemeshow test, which showed no statistically significant evidence of lack of fit (2012, *p* = 0.078, 2019, *p* = 0.135).

## Discussion

Large epidemiological surveys of myopia among schoolchildren in Urumqi remain limited. Tang et al. (9) analysed physical-health-surveillance data from students aged 7–18 years in Urumqi and reported an increase in the detection of reduced visual acuity between 2006 and 2016, although reduced visual acuity is not equivalent to clinically confirmed myopia. In the present study, the crude prevalence of myopia was 39.7% in the 2012 survey and 37.1% in the 2019 survey, while the corresponding equal-weight school-stage-standardised estimates were 45.9 and 43.7%. These values provide two descriptive snapshots of the burden of myopia among surveyed schoolchildren in Urumqi. However, the 2012 and 2019 surveys differed in geographic coverage, sample size, response rate, and school-stage composition. The observed differences between the two surveys may therefore reflect differences in the sampled populations as well as possible changes over time. Accordingly, the present findings cannot establish a population-level temporal trend or demonstrate that the onset of myopia has shifted towards younger ages. Future surveillance using a consistent sampling frame, standardised ophthalmological assessment, and repeated sampling from the same districts is required to evaluate temporal changes more reliably.

Having at least one myopic parent was associated with higher odds of screening-defined myopia in both surveys. Compared with students whose parents had no reported myopia, the adjusted odds were 2.11-fold higher in 2012 and 2.22-fold higher in 2019. These findings are broadly consistent with previous studies showing an association between parental and childhood myopia (15, 16). Previous research has further suggested a gradient according to whether one or both parents are myopic and according to the severity of parental myopia (17, 18). However, parental myopia was available only as a binary variable in the present study, and we were unable to examine such a dose–response pattern.

Familial clustering of myopia may reflect both genetic susceptibility and shared environmental or behavioural characteristics. Although multiple inheritance patterns and genetic loci have been implicated in myopia (19–23), children and their parents may also share patterns of educational exposure, near work, outdoor activity, and other socioeconomic or lifestyle characteristics. Because parental education, household income, and detailed family behavioural information were unavailable, the observed association with parental myopia should not be interpreted as an exclusively genetic effect.

In both the 2012 and 2019 results, this study found that students’ eyes when reading and writing were less than 30 cm away from books and poor reading and writing habits were risk factors for myopia in schoolchildren. Consistent with the results of the present study, Wu et al. (24) concluded against a myopia survey among schoolchildren that reading and writing distances greater than 30 cm were significant protective factors against myopia. In the 2019 survey that frequently looking into distance is a protective factor for myopia. This reason can be explained by the classic theory of accommodation created by Von Helmholtz in 1855, whereby the human eye, when looking at objects that are limited in distance, produces a corresponding contraction in the ciliary muscles that thickens the lens into a convex shape, which enhances the refractive index to enable us to see things that are in close proximity. The eye does not trigger the regulatory function of ciliary muscle when it is in infinity, i.e., in a state of ciliary relaxation, the lens is relatively stretched out and concave (25), whereas the lens of myopic patients has a thicker convex shape. So frequently looking into distance can effectively avoid the occurrence of this phenomenon, thus preventing the occurrence of myopia. Many studies have also confirmed that poor reading and writing posture is a major risk factor for poor vision (26, 27). The poor reading and writing habits include lying to see a mobile phone or reading, holding a pen in the area too lower end or with fingers blocking one eye of sight, skewing of the body or head when writing, read a book when walking or travelling. Because students are closer between eyes and books during reading and writing, if the posture is incorrect at this time, they may further pull closer between eyes and books and thus increase their risk of myopia. In addition, in the wrong reading-writing posture, the child’s head may be biased to one side in order to see more clearly, resulting in one eye being closer to the paper surface and the other more distant. This further leads to large differences in the number of diopters in both eyes, which may also contribute to the development of strabismus.

Reported participation in eye exercises was inversely associated with screening-defined myopia in both the 2012 and 2019 models. However, this observational association should not be interpreted as evidence that eye exercises prevent the onset or progression of myopia. Previous findings have been mixed. Some cross-sectional and cohort studies have suggested modest associations between correctly or frequently performed eye exercises and less myopic refractive status or slower myopic progression (28, 29). In contrast, an urban cross-sectional study found a modest reduction in near-vision symptoms but no clear reduction in myopia (30). A randomised controlled trial found only a small, short-term reduction in accommodative lag, which was considered unlikely to be clinically sufficient to prevent long-term myopic progression (31). The available evidence therefore remains insufficient to establish eye exercises as an effective intervention for preventing myopia.

Several explanations may account for the inverse association observed in the present study. Students who regularly performed eye exercises may have been more compliant with school health routines and may also have followed other recommended behaviours, such as taking breaks from near work, maintaining an appropriate reading distance, or participating in outdoor activities. The scheduled exercise period also provides a brief interruption to continuous near work, which may reduce transient visual fatigue without necessarily affecting refractive development. In addition, the frequency and quality of eye exercises were self-reported, and residual confounding, reporting bias, and reverse causation cannot be excluded. Our findings should therefore be regarded as exploratory and do not demonstrate an independent preventive effect of eye exercises on myopia.

At present, among the findings of numerous scholars worldwide, most support a clear association between time spent outdoors and the development of myopia. A meta-analysis by Ayaki et al. (32) by pooling the results of 7 cross-sectional studies suggested that increasing time spent outdoors may be a strategy to reduce myopia in children and adolescents. This present study similarly found that increasing outdoor activity was a protective factor against myopia in students. The mechanisms underlying the effects of outdoor activity on myopia are not well defined, but there are relevant studies that suggest that light exposure stimulates retinal synthesis and release of dopamine, which shortens the ocular axis (33, 34). The additional increase in time spent outdoors also implies a decrease in the amount of work time spent in the near eye by the pupils, thereby further reducing the risk of myopia.

According to Jung et al. (35), the prevalence of myopia reached 96.5% in urban Korea, including a high myopia prevalence of 21.6%, and reached 83.3% in rural Korea, including a high myopia prevalence of 6.8%. Jee et al. (36) suggested that the reason for this might be related to insufficient sleep. To confirm this contention, they suggested an inverse association between sleep duration and myopia after a cross-sectional study using a systematic, stratified, multistage, cluster based sampling method conducted nationwide in Korea. Retinal circadian rhythms are central to the signalling mechanisms that regulate ocular refractive development, and retinal neurotransmitter responses interact with the retinal circadian clock to control the circadian rhythm of eye growth and size, thereby regulating ocular chemotactic development (37). In addition, we speculated that long-term occupation of student sleep time with near eye work might be one of the reasons for the lack of students’ sleep. Insufficient sleep or sleep disturbance may therefore contribute to the rising risk of myopia in students.

The associations with computer use and television viewing differed between the two surveys. Daily computer use was associated with higher odds of screening-defined myopia in 2012, although the estimate was borderline, whereas the corresponding association was not statistically significant in 2019. Conversely, a higher television-viewing category was inversely associated with screening-defined myopia in the 2019 model. These findings should be interpreted cautiously. The two surveys differed in sampling frame and participant composition, and the year-specific models were not intended to estimate changes in the effects of individual screen-based activities over time.

The inverse association with television viewing was unexpected and should not be interpreted as evidence that television viewing protects against myopia. Television use may represent differences in how children allocate their leisure time, but it may also be associated with age, school workload, socioeconomic circumstances, outdoor activity, near work, or other unmeasured behaviours. The questionnaire did not objectively measure viewing distance, simultaneous use of multiple devices, or total screen exposure. Moreover, comparable quantitative data on mobile-phone and tablet use were unavailable. We therefore cannot determine whether changes in the use of mobile devices contributed to the different computer- and television-related findings between the two surveys. Future studies should assess total screen exposure, individual device use, viewing distance, and continuous near-work duration using harmonised and preferably objective measures.

The 2019 findings also showed that eyes rest after 1 h of reading and writing time is a protective factor for myopia, which is consistent with Wu et al. (24) research. The 2019 survey showed an inverse association between eating zhuafan more than twice per week and screening-defined myopia. However, this association was not statistically significant in the 2012 model, which limits its consistency across the two surveys. Moreover, zhuafan is a mixed dish whose ingredients, portion size, and preparation methods may vary substantially between households. The questionnaire measured only weekly consumption frequency and did not assess portion size, nutrient intake, total energy intake, other foods, or overall dietary patterns. Consequently, the observed association cannot be attributed to carrots, vitamin A, or any other specific nutrient. Frequency of zhuafan consumption may also represent unmeasured differences in ethnicity, household socioeconomic circumstances, meal regularity, family dietary practices, or other lifestyle characteristics. Despite adjustment for the available covariates, residual confounding remains possible. This finding should therefore be considered exploratory and hypothesis-generating. Studies incorporating validated dietary assessment methods and detailed nutrient information are needed before any dietary or biological interpretation can be made.

This study has several limitations. First, the 2012 and 2019 surveys were independent cross-sectional surveys based on different sampling frames. The 2012 survey included Xinshi and Shayibake districts, whereas the 2019 survey additionally included Tianshan and Shuimogou districts. The surveys also differed substantially in sample size, response rate, and school-stage composition. An analysis restricted to the two districts common to both surveys could not be performed because the individual-level historical information required to reconstruct harmonised district-specific samples was unavailable. Although internally standardised prevalence estimates were calculated using equal weights across the prespecified age and school-stage strata, this procedure could not eliminate differences in geographic coverage or other characteristics of the sampled populations. Consequently, the observed between-survey differences should be interpreted descriptively and not as definitive evidence of a population-level temporal trend. Second, the study outcome was based on an operational screening definition using uncorrected distance visual acuity <5.0 in at least one eye, followed by an ophthalmic examination to exclude clinically identifiable alternative ocular conditions. Cycloplegic refraction and spherical equivalent measurements were not available. Pupil dilation and ophthalmic examination cannot substitute for cycloplegic refraction when determining refractive status, and some students may therefore have been misclassified. Accordingly, the reported estimates represent the prevalence of screening-defined myopia and may not be directly comparable with estimates based on spherical equivalent thresholds following cycloplegic refraction. Third, the response rate was 80.8% in 2012, compared with 95.0% in 2019. Demographic, visual, and behavioural information on non-responders was unavailable, precluding a formal non-response analysis. If participation was associated with visual status, school attendance, health awareness, or relevant behavioural characteristics, selection bias may have affected the prevalence and association estimates. The direction and magnitude of this potential bias could not be determined. Fourth, several behavioural and environmental variables were self-reported, including reading and writing habits, outdoor activity, sleep duration, eye exercises, and computer and television use. These measurements may have been affected by recall error, social-desirability bias, and exposure misclassification. Information on the accuracy, duration, supervision, and quality of eye exercises was unavailable. Comparable quantitative data on mobile-phone and tablet use were also unavailable, and the study did not measure total screen exposure, viewing distance, simultaneous use of multiple devices, or uninterrupted near-work duration. Classroom lighting and other environmental conditions were assessed subjectively rather than measured directly. Fifth, several potentially important confounders were not consistently available across the two surveys, including parental educational attainment, household income, individual-level urban–rural residence, school workload, and detailed parental myopia status. Parental myopia was available only as a binary variable indicating whether at least one parent had myopia; the study could not distinguish between one and two myopic parents or account for the severity of parental myopia. Dietary assessment was limited to the reported weekly frequency of eating zhuafan and did not capture portion size, ingredients, preparation methods, nutrient intake, total energy intake, or overall dietary patterns. Residual confounding by socioeconomic circumstances, residential environment, familial behaviours, cultural factors, and other unmeasured characteristics therefore cannot be excluded. Sixth, the cross-sectional design precluded determination of the temporal sequence between the identified factors and screening-defined myopia. Candidate behavioural and environmental variables were selected for the multivariable models using a year-specific univariable *p*-value threshold of 0.05. Although sex, ethnicity, age group, and school stage were included *a priori* in both models, the complete sets of candidate variables were not identical. Therefore, adjusted ORs from the two models should not be compared directly as evidence of changes in the strength of associations between 2012 and 2019. All regression findings should be interpreted as exploratory associations rather than causal effects. Finally, the study was restricted to schoolchildren in Urumqi, which may limit the generalisability of the findings to populations with different socioeconomic, environmental, educational, or cultural characteristics.

## Conclusion

Combined with the results of this program and our research, prevention and control of myopia in adolescents can start with increasing the time spent outdoors, controlling the use of electronic products by students, reasonably arranging and stimulating students to do eye exercises, reasonably arranging the time spent with eyes, correcting students’ poor reading and writing habits, reasonably formulating dietary structure, conducting regular vision testing efforts, and strengthening health education. A scientific prevention and control working group that forms the ‘Trinity’ of education, scientific research and medical and health authorities should aim to promote the establishment of a pilot zone for myopia prevention and control reform in Urumqi more rapidly and more effectively reduce myopia rates among schoolchildren.

## Data Availability

The raw data supporting the conclusions of this article will be made available by the authors, without undue reservation.
